# Enhanced adsorption and removal of Cd(II) from aqueous solution by amino-functionalized ZIF-8

**DOI:** 10.1038/s41598-024-59982-9

**Published:** 2024-05-10

**Authors:** Amir Khosravi, Razieh Habibpour, Maryam Ranjbar

**Affiliations:** Department of Chemical Technology, Iranian Research Organization for Science and Technology (IROST), Tehran, Iran

**Keywords:** ZIF-8, Post modification, Ethylenediamine, Adsorption, Cadmium, Environmental sciences, Environmental chemistry

## Abstract

Zeolite imidazolate framework-8 (ZIF-8), which is a special subgroup of metal–organic frameworks (MOFs), was synthesized and modified by ethylenediamine (ZIF-8-EDA) to prepare an efficient adsorbent for the high sorption of Cd^2+^ ions from solution. The synthesized and modified ZIF-8 (ZIF-8-EDA) were characterized by X-ray diffraction (XRD), Fourier-transform infrared (FT-IR) spectroscopy, Brunauer–Emmett–Teller (BET), field emission scanning electron microscopy (FE-SEM) with energy dispersive spectroscopy (EDS), and transmission electron microscopy (TEM) analysis. The optimum conditions for dosage of adsorbent, initial ion concentration, pH, and contact time were 0.05 g/l, 50 mg/l, 6, and 60 min, respectively, for cadmium ion sorption from aqueous solutions with a removal efficiency of 89.7% for ZIF-8 and 93.5% for ZIF-8-EDA. Adsorption kinetics and equilibrium data were analyzed using the Langmuir and Freundlich equations. The Langmuir model fitted the equilibrium data better than the Freundlich model. According to the Langmuir equation, the maximum uptake for the cadmium ions was 294.11(mg/g). The calculated thermodynamic parameters (ΔG°, ΔH°, and ΔS°) indicated that the adsorption process was feasible, spontaneous, and endothermic at 20–50 °C. Based on the results, the amino functionalized ZIF-8 had improved adsorption performance due to the replacing of the starting linker with organic ligands that had effective functional groups, leading to chemical coordination due to the interaction of metal ions with the non-bonding pair of electrons on the N atoms of the amino functional group. The selectivity toward metal ion adsorption by ZIF-8-EDA was Cd^2+^ > Pb^2+^ > Ni^2+^‏.

## Introduction

The fast development of industry and agriculture has significantly polluted the environment due to water contaminants. Among the causes of water pollution, heavy metals are extremely toxic to humans^1–3^. Within heavy metals, cadmium (Cd) has high acute toxicity and is dangerous to humans, animals, and plants. It can be introduced into the environment through wastewater from various industries, including non-ferrous metals, electroplating, electronics, pigments, dyes, textiles, and alkaline battery refining and manufacturing^4^. Under high doses of cadmium, the central nervous system, kidneys, liver, lungs, bones, and cardiovascular system suffer acute or chronic damage^5–7^. Conventional methods of removing aqua heavy metal ions include ion exchange, adsorption, chemical precipitation, electrochemical separation, etc.^8–11^. Excluding adsorption, all these methods suffer from limitations: high consumption of reagents and energy, high formation of secondary contaminants, low selectivity, and slow kinetics. The adsorption process has many advantages, including high efficiency, simple design and operation, low cost, and high removal selectivity at very low concentrations of the metals^12,13^.

During the last decade, MOFs have been widely used as a promising absorbent material for aqua heavy metal ion removal. They are produced by bridging organic ligands as linkers and metal ions through self-assembly synthesis. They can be designed and fabricated using cost-effective and flexible synthetic methods with high porosity, uniform pore size distribution, and geometries that preserve the desired compounds with high yields^14,15^. These properties have increased their use in numerous research fields, such as gas separation and purification, heterogeneous catalysts, photocatalytic and adsorption reactions, and organic chemical separation^16–18^. As a special subclass of MOFs, Zeolitic imidazolate frameworks (ZIFs) are widely used to remove aqua heavy metal ions due to zeolite-like characteristics, such as large specific surface area, good porosity, narrow and size-tunable pore size, and remarkable resistance to chemical or thermal changes^19–21^. ZIF-8 is a member of this class because it is coordinated tetrahedrally by Zn^2+^ as a metal ion and 2-methylimidazole as a linker.

Post-synthetic modification (PSM) is a powerful approach for preparing new MOFs, which has considerable scope in exploiting many application fields of the MOFs without altering the primary topology and bond structure of the compound. The MOF specific surface environment can be improved to reinforce the chemical-thermal stability of the structure and introduce desirable properties. Post-synthetic modifications can be performed through methods like linker-metal node adapting, adsorption, or host–guest exchange. Hence, functionalization of the mesoporous channels’ surface in the MOFs is an effective strategy to increase their adsorption capacity for various molecules^22–25^. Post-modification of MOFs with different functional groups is a significant area of research which demands a careful approach to the design and execution of various fields. It is promising and widely used as an effective technique to upgrade the adsorption properties of MOFs.

Wang et al.^26^ reported that Cu_3_(BTC)_2_–SO_3_H, a functionalized MOF by sulfonic acid, displayed high removal efficiency for cadmium ions from aqueous solution after functionalization. Binaeian et al.^27^ employed the facile method to prepare ZIF-8 modified by dimethylethylenediamine for the sorption of ${Cd}_{\left(aq\right)}^{2+}$ metal ions with a removal efficiency of 85.38%. Roushani et al.^28^ synthesized and applied a new functional MOF (TMU-16-NH_2_) for the rapid extraction of ${Cd}_{\left(aq\right)}^{2+}$ metal ions. The maximum removal efficiency was 98.91% at 30 min contact time and pH = 6.0. Luo et al.^29^ used a PSM tactic to obtain thiol-functionalized magnetic MOFs for quick and discerning adsorption of aqueous Hg and Pb metal ions with large amounts of coexisting ions. Wang et al.^30^ synthesized Zr-MOFs functionalized by amino group by fast microwave-assisted method for use in the adsorption of aqueous Pb and Cd metal ions; they noted that the adsorption magnitude of ${Cd}_{\left(aq\right)}^{2+}$ was ~ 177 mg/g. According to this research, functionalized MOF may have a better adsorption effect on heavy metal ions such as cadmium in aqueous solutions compared to the parent frameworks.

In this study, modified ZIF-8 by ethylenediamine (ZIF-8-EDA) was used as a high-potential adsorbent. It was synthesized with the solvothermal method and applied to investigate the adsorptive removal of Cd (II), as the target pollutant, from aqueous solution in comparison to pure ZIF-8, which is presented in detail in our previous work^31^. The properties of synthesized ZIF-8-EDA adsorbent were studied using XRD, FT-IR, FE-SEM, TEM, EDS, and BET techniques. Furthermore, the effects of principal factors, such as pH, ion concentration, adsorbent dosage, and contact time, on adsorption performance were well elucidated using batch adsorption methods. Also, adsorption was modeled using the Langmuir and Freundlich isotherm models. The adsorption mechanisms of cadmium ions onto ZIF-8-EDA were also evaluated in terms of thermodynamics and kinetics.

## Experimental section

### Materials and methods

All chemicals and reagents, e.g., Zn(NO_3_)_2_.6H_2_O, DMF (dimethylformamide), 2-methylimidazole, ethylenediamine (EDA), chloroform, and anhydrous hexane, were obtained from Merck and used without any further purification. The ZIF-8 adsorbent was prepared according to the procedures defined in our earlier study^31^. Initially, 1.88 g of Zn(NO_3_)_2_.6H_2_O was dissolved in 50 mL of DMF solvent. Afterward, the mentioned solution was placed rapidly in a hydrothermal autoclave reactor containing a solution of 2-methylimidazole. Subsequently, the reaction was completed in an autoclave at 140 °C in the oven. After cooling, the obtained powder of ZIF-8 was isolated from the solution by centrifuging; then, it was washed three times with chloroform. Eventually, the remaining solvent was dried at 200 °C in a vacuum for 6 h to yield 0.2 g of white crystals. The amino-functionalized ZIF-8 with ethylenediamine was prepared to conform to the method used by Miralda et al.^32^. For this functionalization, 2.0 g of ZIF-8 powder was combined with 30 mL of anhydrous hexane. The suspension was inserted into 13 mL of EDA and refluxed at 50 °C for 20 h under N_2_ gas to complete the effective linking of the functional group. Finally, the prepared light-yellow powder was obtained by centrifugation and washed with hexane. Before use, the modified adsorbent (ZIF-8-EDA) was activated by drying at 60 °C for 24 h.

### Adsorption studies

Batch adsorption experiments were used for aqua cadmium ion elimination and the effect of fundamental factors, e.g., starting metal ion concentration, dosage of adsorbents, pH, and contact time, on the sorption performance of ZIF-8 and ZIF-8-EDA adsorbents. For each experiment, 100 ml of an aqueous solution containing a known concentration of metal ions was inserted in an Erlenmeyer, including a specified amount of adsorbent. These samples were stirred in a shaker at 300 rpm under constant temperature. In this procedure, the starting metal ion concentration was set between 10 and 60 mg/L. The effects of the amount of adsorbent, solution pH, and contact time on the sorption function were studied and optimized within ranges of 0.01–0.06 g/L, pH of 3–9, and 5–120 min, respectively. The final aqueous solutions were separated from the adsorbent by centrifuging at 3000 rpm for fifteen minutes and then examined via atomic absorption spectrometry (AAS) to determine the amount of metal ions. The percentage of removal efficiency was achieved using Eq. 1.

1 $$\mathrm{Removal \,performance }(\mathrm{\%}) =\frac{{C}_{i}-{C}_{e}}{{C}_{i}} \times 100$$

where C_i_ and C_e_ are the metal ions concentrations (mg/L) in the solution at the beginning of the adsorption and at the equilibrium conditions, respectively.

### Adsorbent regeneration studies

Regeneration studies of Cd^2+^ ions from the ZIF-8-EDA adsorbent were carried out by shaking the loaded adsorbent after adsorption/desorption equilibrium in HCl solutions (0.1 mol/L) as the desorbing agent for 2 h at room temperature. Then, the desorbed ZIF-8-EDA adsorbent was separated from the acid solution, washed with excess distilled water three times, and dried. Afterward, the adsorbent was reused repeatedly to remove Cd^2+^ from aqueous solutions in the subsequent experiments under the same conditions.

## Result and discussion

### Metal organic framework structure, morphology, and porosity evaluation

**Figure 1:**
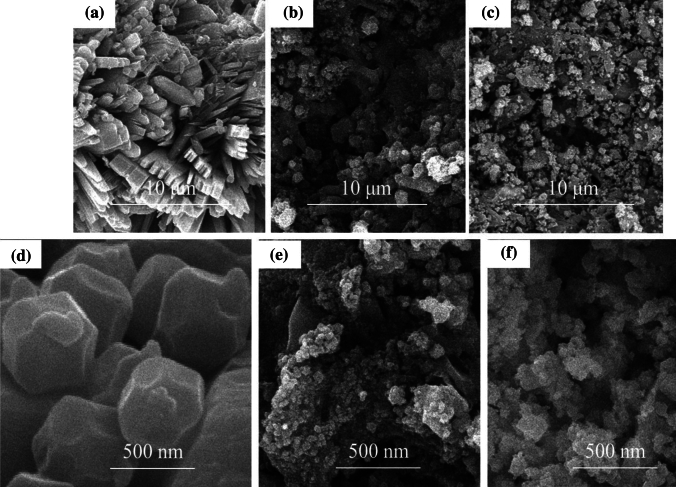
FE-SEM pictures of synthesized ZIF-8 (**a** and **d**), ZIF-8-EDA (**b** and **e**), and ZIF-8-EDA after ${Cd}_{\left(aq\right)}^{2+}$ ions adsorption (**c** and **f**).

The porosity size distribution measurement and microstructure of the synthesized ZIF-8 and ZIF-8-EDA under cadmium metal ion adsorption were detected by FE-SEM (Tescan VEGA3; accelerating voltage: 200 V to 30 kV) to investigate the surface morphology. As shown in Fig. 1a,d, the truncated octahedral crystals of ZIF-8 are obvious and have many hollow spaces in their construction capable of uptaking ${Cd}_{\left(aq\right)}^{2+}$ ions. After modification with EDA, the structure of the amino functionalized ZIF-8 tended to be rougher and irregular with smaller particle sizes, indicating that the attachment of new ligands made it possible to obtain the new morphology and structure in the compounds (Fig. 1b,e). The sorption of the cadmium ions greatly reduced the particle size and strongly affected the morphology, while the truncated octahedral crystal structure was still maintained (Fig. 1c,f). Based on these images, it could be said that functionalization and adsorption strongly affected the morphology, creating higher porosity and smaller particles without changing the crystal structure.

**Figure 2:**
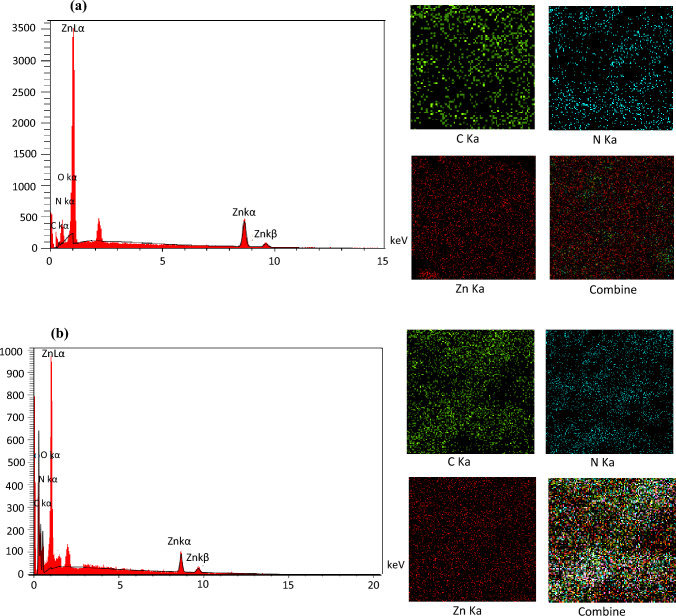
EDS spectrum and elemental mapping of Zn, N, and C of ZIF-8 (**a**) and ZIF-8-EDA (**b**).

The elemental composition and chemical characterization of ZIF-8 and ZIF-8-EDA were studied via EDS as an X-ray technique. The existence of C, N, and Zn elements on the surface of ZIF-8-EDA and ZIF-8 can be observed in Fig. 2. Moreover, the elemental mapping analysis established that N, C, and Zn were dispersed evenly near and on the adsorbent surface, as indicated by the EDS data. The nitrogen and oxygen elements found in the construction of ZIF-8 were from the 2-methylimidazole ligand. In ZIF-8-EDA, in addition to the 2-methylimidazole ligand, the N atoms came from the ethylenediamine ligand^33^. After the amino-modification of ZIF-8, the most apparent changes occurred in the form of a decrease in the amount of zinc content and an increase in nitrogen content, which indicated the loading of ZIF-8 by ethylenediamine.

**Figure 3:**
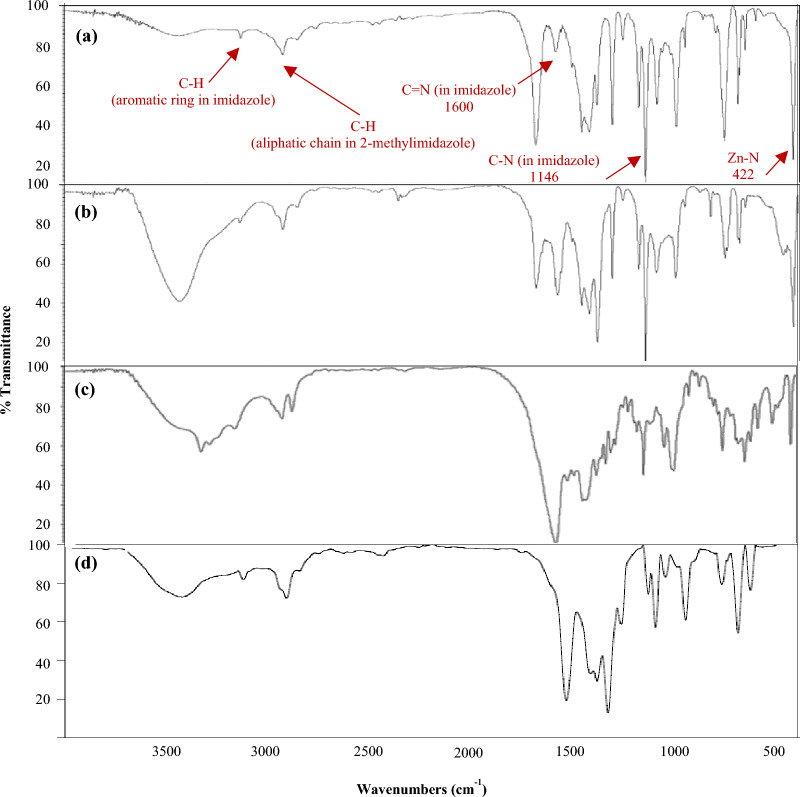
FTIR spectra of bare ZIF-8 (**a**), ZIF-8 after ${Cd}_{\left(aq\right)}^{2+}$ ions adsorption (**b**), bare ZIF-8-EDA (**c**), and ZIF-8-EDA after ${Cd}_{\left(aq\right)}^{2+}$ ions adsorption (**d**).

FT-IR (Thermo Nicolet Avatar, USA) analyses were used to analyze the adsorptive mechanisms and the functional segments of ZIF-8 and ZIF-8-EDA, before and after cadmium ions adsorption. As presented in Fig. 3a, the peaks at 3131 cm^−1^ and 2921 cm^−1^ were from the stretching vibrations of aromatic and aliphatic C–H bonds of the imidazole ring, respectively. The stretching of the C=N bond was observed at 1600 cm^−1^. The vibrations (both bending and stretching) of the imidazole ring appeared at 600–1500 cm^−1^. The stretching vibration of the Zn–N bond appeared at 422 cm^−1^ and indicated that Zn atoms were linked to the N atoms of the 2-methylimidazole within the preparation of the ZIF-8. According to the existence of an imidazole linker and Zn–N coordination bond in the FT-IR spectra, the crystallinity of the prepared ZIF-8 was in good agreement with previous studies^34–36^. After the adsorption of ${Cd}_{\left(aq\right)}^{2+}$ ions by ZIF-8 (Fig. 3b), new peaks at 540 and 139 cm^−1^ appeared that were related to Cd–N stretching vibrations, indicating that Cd^2+^ had been bonded onto ZIF-8 through an N atom on the 2-methyleimidazole linker^37^. The presence of adsorption peaks from 2800 to 3200 cm^−1^ indicated the retention of the ZIF-8 construction during cadmium ion removal. In addition, the ZIF-8 framework's preservation during cadmium ion removal was indicated by the weakening of the peaks between 2800 and 3200 cm^−1^, suggesting that adsorption was the primary process for removing cadmium ions.

The FT-IR analysis of ZIF-8-EDA is displayed in Fig. 3c. As shown, an absorbance peak at 3325 cm^−1^ represented the coupling of the N atom in the ethylenediamine group with an unsaturated Zn center in ZIF-8-EDA and confirmed the loading of ethylenediamine into ZIF-8^38,39^. Also, a peak of about 900 cm^−1^ appeared after Cd^+2^ ion adsorption on ZIF-8-EDA (Fig. 3d), confirming that an electron lone pair of N atoms interacted with the Cd^2+^ ions. During the process of removing cadmium, the surface of the synthesis adsorbent also experienced sorption and ion exchange interaction. This was authenticated by the existence of a wide peak from 3300 to 3800 cm^−1^, which could be attributed to zinc oxide.

**Figure 4:**
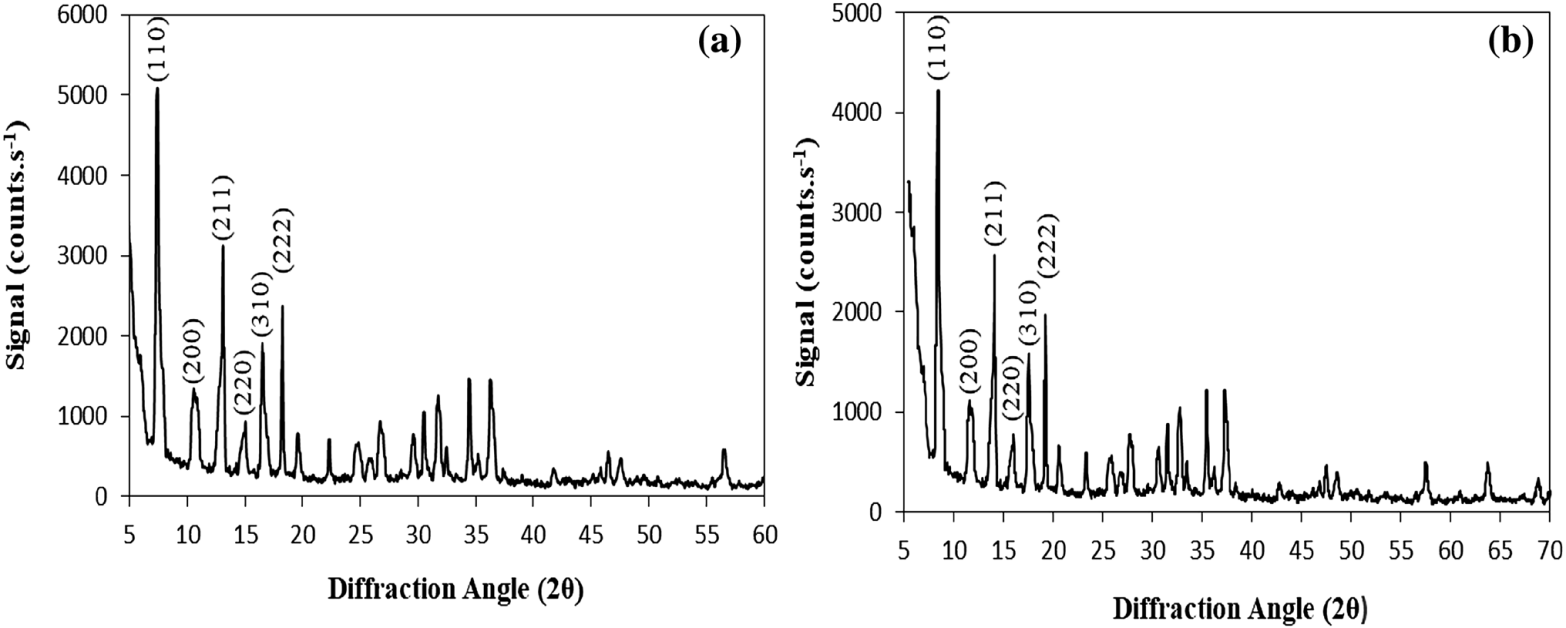
XRD graphs of ZIF-8 (**a**) and ZIF-8-EDA (**b**).

Using XRD analysis, the crystalline structure of ZIF-8 and ZIF-8-EDA was determined, and their synthesis was confirmed. In Fig. 4a, the main peaks at 2θ = 7.4, 13.05, 16.55, 18.15, and 26.5 followed the presented patterns and proved that ZIF-8 was successfully synthesized^40,41^. Furthermore, the presence of a sharp peak at 2θ = 7.4 (below 10°) indicated the high crystallinity of the synthesized ZIF-8. The XRD patterns of ZIF-8-EDA (Fig. 4b) were extremely close to those of ZIF-8, except for some minor changes in the peak intensities because of the partially loaded pores of ZIF-8 by ethylenediamine. Actually, the EDA functional segment masked the internal and external surface of ZIF-8 and led to decreases in the peak intensities without affecting its crystalline structure. It indicated that the presence of ethylenediamine as a functional group did not destroy or decompose the ZIF-8 structure but was also a synthesized, well-ordered ZIF-8-EDA^42,43^.

BET analyses were applied to measure the surface area, total pore volume, and mean pore diameter of the adsorbents (Table 1). Based on these results, the specific surface area of ZIF-8-EDA increased from 327.37 to 404.46 m^2^/g with respect to the bare ZIF-8. It probably occurred because of the additional amount of organometallic framework used for modification. Furthermore, the amino functional group was well coupled to the zinc-centered active site and the ligand, which caused an increase in surface areas. Also, the findings indicated that the total pore volume improved after the charging of ZIF-8 by ethylenediamine. The setup of additional micropores at the interface was the main reason for this behavior, which was essential for the metal ions migration from aqua solutions into the pores, resulting in their adsorption^39,44^.

**Table 1:** Surface area and porosity data of ZIF-8 and ZIF-8-EDA.

Adsorbent	BET surface area (m^2^/g)	Langmuir surface area (m^2^/g)	Mean pore diameter (nm)	Total pore volume (cm^3^/g)
ZIF-8	327.37	303.38	1.7513	0.1328
ZIF-8-EDA	404.46	377.92	1.9752	0.1997

**Figure 5:**
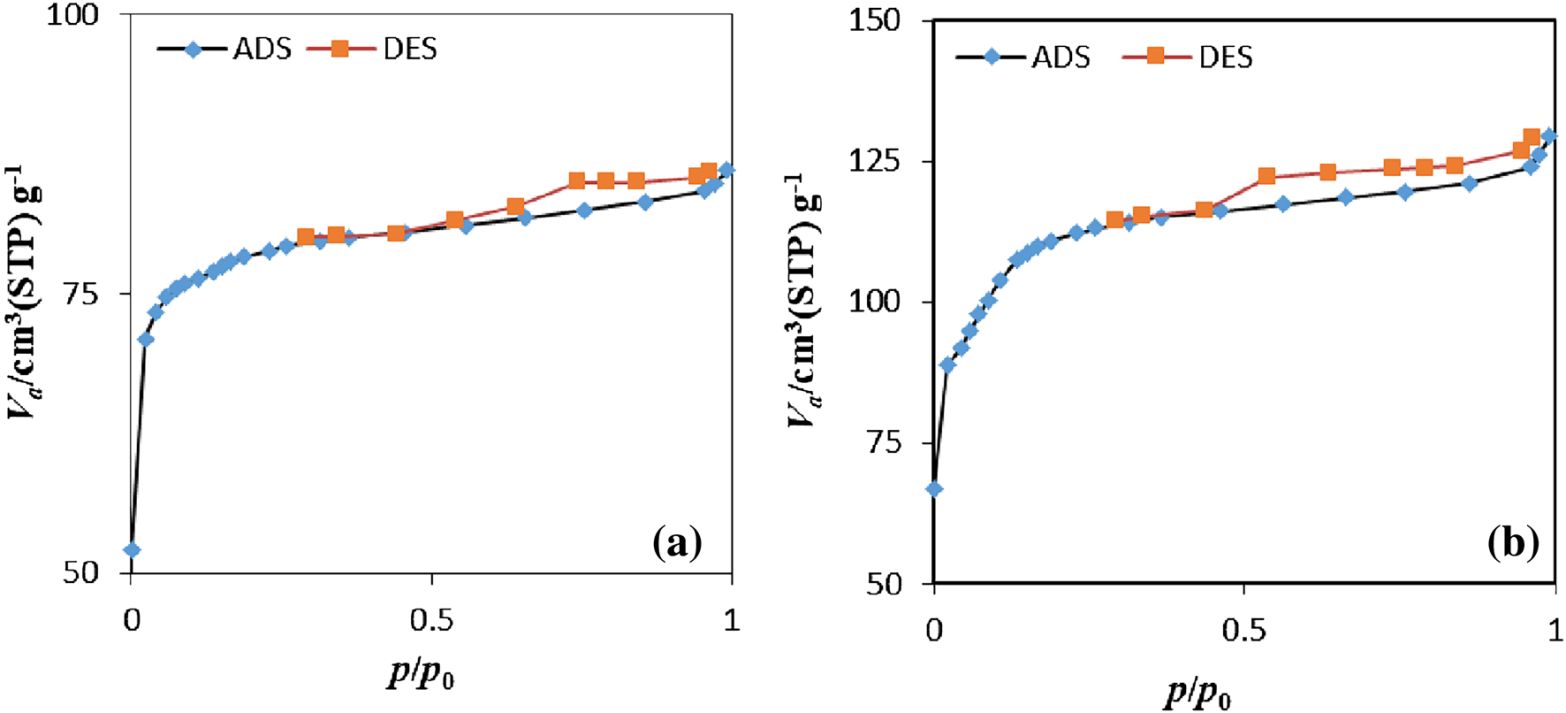
N_2_ adsorption/desorption isothermal diagrams of ZIF-8 (**a**) and ZIF-8-EDA (**b**).

The surface area and porosity data of ZIF-8 and ZIF-8-EDA were measured based on N_2_ adsorption/desorption isotherms at 77 K. To obtain an adsorption/desorption isotherm, different relative pressures were used to calculate the amount of adsorbed gas at a constant temperature. The obtained N_2_ adsorption/desorption isotherm for both adsorbents was consistent with a typical type I adsorption isotherm (Fig. 5); there is a very small step with a hysteresis loop at high pressure, demonstrating the presence of small mesopores in their crystal structure^45^. However, the adsorbed nitrogen amount of ZIF-8 was greater than that of ZIF-8-EDA at each relative pressure because the pores and inner surface of ZIF-8 were loaded by ethylenediamine.

**Figure 6:**
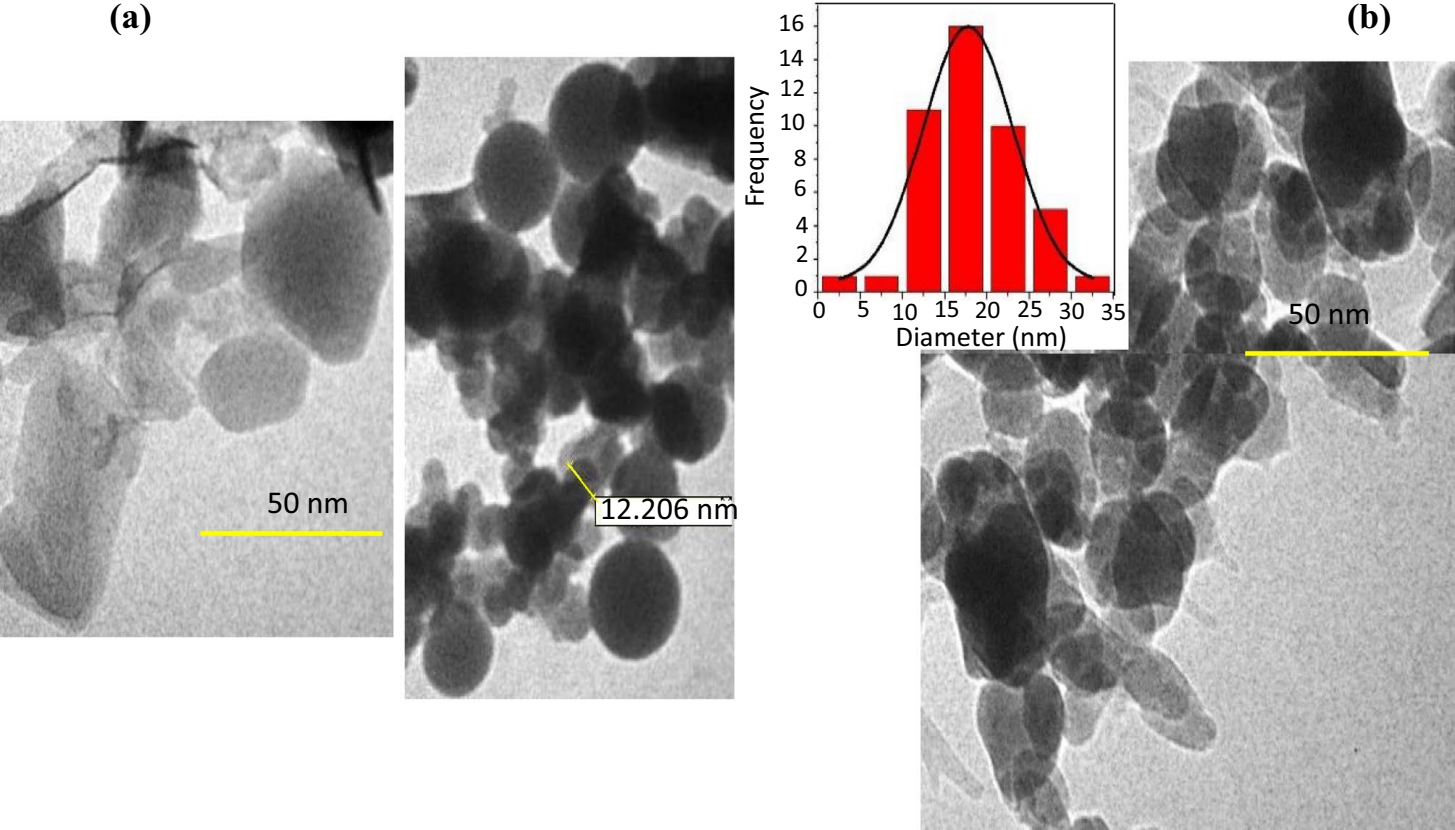
TEM pictures of ZIF-8 (**a**) and ZIF-8-EDA (**b**).

TEM was used as a useful analytical technique to characterize the structure, give a picture of the bulk, and display the three-dimensional form of prepared adsorbent crystallites. The TEM images and size distribution histograms of these samples are depicted in Fig. 6, where it is possible to recognize the nano-sized particles and sharp hexagonal faces. The crystallite’s diameter for ZIF-8 was 12–30 nm, which was consistent with the FE-SEM observations (Fig. 1a,d)^34,46^. Figure 6b shows that the surfaces of the amine-functionalized ZIF-8 tended to be rougher and became more agglomerated after modification with EDA, and the particle size was smaller. This clearly indicated that the amine post-modification was loaded onto ZIF-8.

### Adsorption experiments

**Figure 7:**
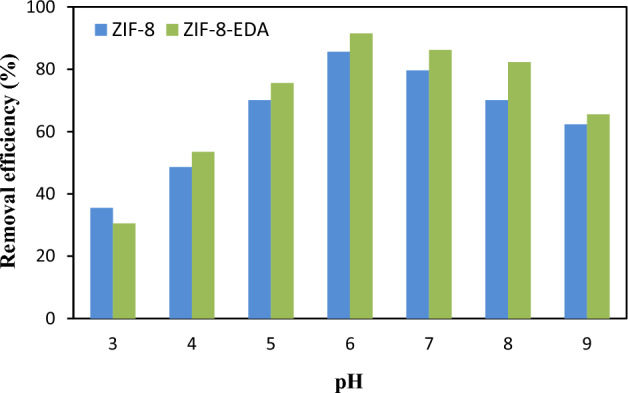
Effect of pH on ${Cd}_{\left(aq\right)}^{2+}$ ions sorption by adsorbents (settings: [metal ion]_i_ = 50 mg/L; adsorbent dosage = 0.05 g/L; contact time = 60 min).

The optimum adsorption characteristics of ZIF-8 and ZIF-8-EDA for Cd^2+^ ions from aqueous solution were performed in the batch scale. Ion concentration, pH of solution, dosage of adsorbent, and contact time were investigated as effective parameters in the sorption of ${Cd}_{\left(aq\right)}^{2+}$. The pH of solution with the change of the metal ions speciation, as well as the structural stability of the absorbent and its surface properties, had an important effect in the sorption of metal ions from aqua environments. In this investigation, the solution’s pH values, ranging from 3 to 9, were investigated. Figure 7 indicates that as pH increased, the adsorption efficiency of Cd^2+^ ions significantly improved; the maximum efficiency was reached at pH = 6.0, where more than 82% and 85% of Cd^2+^ ions could be removed using ZIF-8 and ZIF-8-EDA, respectively. At a lower pH, the amount of hydronium ions (H_3_O^+^) increased, and for this reason, its competition with positive amine groups (NH_2_^+^) of ethylenediamine increased. In this condition, positive Cd^2+^ ions approached the negative surface of the adsorbent, which led to a decrease in Cd^2+^ ion elimination. By increasing the pH of the solution, more vacant sites were exposed for the adsorption of Cd^2+^ ions, and as a result, the elimination efficiency increased. This behavior could be elucidated by reducing the concentration of H_3_O^+^ and NH_2_^+^ ions in the solution and then reducing the electrostatic barrier between the Cd^2+^ ion and adsorbent^29^. When the pH increased, the free amine functional segments interacted strongly with Cd^2+^ ions by exchanging valence electrons, leading to a higher rate of ion adsorption. As pH increased to 6, the surface's pretension and positive charge density decreased, causing an increase in the adsorption of Cd^2+^ ions due to electrostatic attraction^47,48^. As can be observed, the removal performance of the adsorbent decreased at pH > 6 due to the precipitation of ion hydroxide that may be formed with Cd^2+^ ions over the adsorption of the adsorbents. Therefore, a pH of 6 was kept constant as the optimum value for the following experiments.

**Figure 8:**
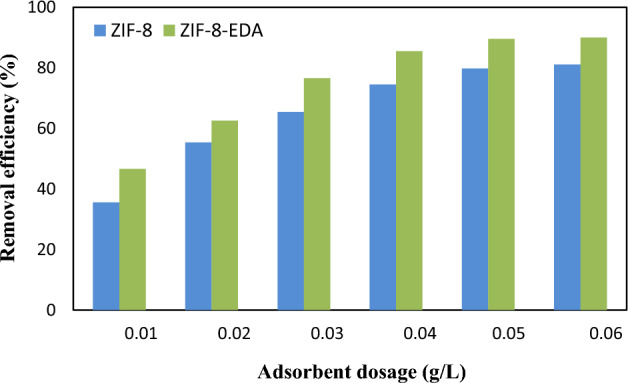
Effect of adsorbent dosage on ${Cd}_{\left(aq\right)}^{2+}$ sorption by adsorbents (settings: [metal ion]_i_ = 50 mg/L; pH = 6.0; contact time = 60 min).

The adsorbent dosage was studied to determine its effect on cadmium ion removal between 0.01 and 0.06 g/L, with a beginning metal ion amount of 50 mg/L and pH = 6 for 60 min. The sorption process was carried out on the surfaces of the adsorbent; the adsorbent characteristics, such as the cavity shape, porosity, and surface area, significantly affected the adsorption efficiency. Due to the increase in the quantity of adsorption active sites and total surface area, increasing the adsorbent dosage promotes an increase in the adsorption efficiency of the metal ions^49,50^. As shown in Fig. 8, adsorption did not increase remarkably when the adsorbent dosage reached more than ~ 0.06 g/L. The reason for this was the accumulation of absorbent particles, which was associated with a decrease in their active surface adsorption sites. Hence, the adsorbent dosage of 0.05 g/L was kept constant as the optimal amount for subsequent Cd^2+^ ion removal experiments. The lack of dispersion during the sorption process in the aqueous solutions caused ZIF-8 to have less efficiency than ZIF-8-EDA. In addition, according to the BET analysis, the ZIF-8-EDA structure had more surface area, so an increase occurred in the cadmium ion removal percentage.

**Figure 9:**
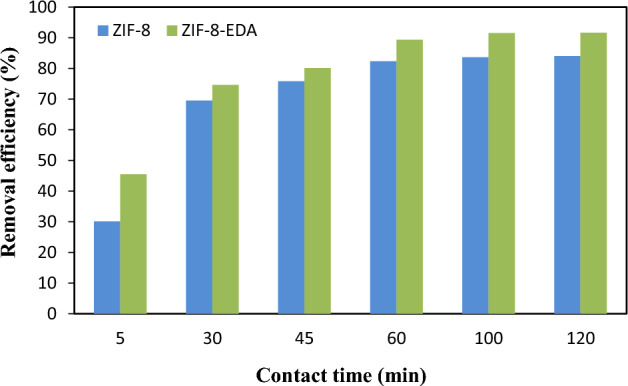
Effect of contact time on ${Cd}_{\left(aq\right)}^{2+}$ sorption by adsorbents (settings: [metal ion]_i_ = 50 mg/L; pH = 6.0; and adsorbent dosage = 0.05 g/L).

In this section, the effect of contact time between 5 and 120 min on the adsorption of Cd^2+^ ions using synthetic adsorbents was investigated. Figure 9 shows that the removal efficiency of Cd^2+^ increased quickly with rising contact time until the equilibrium state. Excess cadmium adsorption was not observed after this time (60 min) due to the absence of an accessible active site, resulting in reduced removal rate^51,52^. Therefore, it could be said that the equilibrium time of Cd (II) ion absorption was 60 min for both adsorbents.

**Figure 10:**
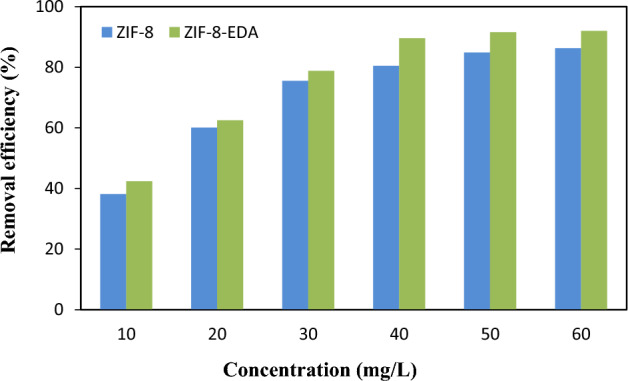
Effect of [${Cd}_{\left(aq\right)}^{2+}$]_i_ on removal efficiency by adsorbents (settings: pH = 6.0; adsorbent dosage = 0.05 g/L; contact time = 60 min).

The initial metal ion concentration had an important effect on the removal performance of the adsorbents. Figure 10 shows the effect of [M^n+^]_i_ = 10–60 mg/L. As shown, increasing [M^n+^]_i_ via an increase in the pressure gradient concentration that also intensified the driving force led to an increase in adsorption. In situations with a low initial metal ion concentration, the amount of ${Cd}_{\left(aq\right)}^{2+}$ ions was reduced compared to the active adsorption sites on the adsorbent surface. The obtained results show that 84.01% and 86.95% of Cd^2+^ ions were derived by ZIF-8 and ZIF-8-EDA, respectively. The results indicated that the removal percentage did not change significantly at a beginning metal ion concentration of more than 50 mg/L. This observation could be obtained when the adsorption was nearer to the saturation state because of the limited available sites. Therefore, 50 mg/L was selected as the optimum ion concentration for the adsorption of Cd^2+^ ions.

**Figure 11:**
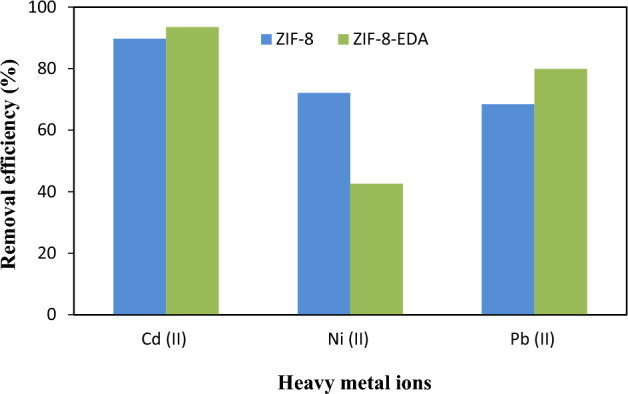
Comparison of removal efficiency (%) of Cd^2+^, Ni^2+^, and Pb^2+^ metal ions by pure ZIF-8 and functionalized ZIF-8-EDA (settings: pH = 6.0; [metal ion]_i_ = 50 mg/L; adsorbent dosage = 0.05 g/L; contact time = 60 min).

The selective adsorption of target heavy metals by MOFs is largely dependent on functional groups. Hence, pure ZIF-8 and its functionalized derivative, e.g., ZIF-8-EDA, were evaluated for the adsorption of Cd^2+^ ions in comparison with Ni^2+^ and Pb^2+^ ions using a 0.05 g/L adsorbent dosage in 100 mL of aqueous solutions with 50 mg/L metal ion concentrations at pH = 6.0 for 60 min. Pb(NO_3_)_2_.6H_2_O, Ni(NO_3_)_2_·6H_2_O, and Cd(NO_3_)_2_·6H_2_O were dissolved in DI- water to prepare a stock solution of metal ions. Figure 11 shows that the removal efficiency of the Pb^2+^, Ni^2+^, and Cd^2+^ ions was 68.43%, 70.1%, and 85.7% by pure ZIF-8 and 79.9%, 45.5%, and 91.5% by ZIF-8-EDA, respectively. Consequently, ZIF-8-EDA, with superior selective sorption capacity, was capable of removing Cd^2+^ ions with more efficiency than the other two metal ions.

The theory of Hard-Soft-Acid–Base (HSAB) is the basis for explaining the selective adsorption of Cd^2+^ ions using ZIF-8-EDA. According to this concept, the soft–soft and hard-hard interactions in the reactants are more stable than the soft-hard interactions in the products^53–55^. Cadmium ions with free orbitals in their electronic structure are classified as soft acid due to their more polarizable and larger ionic radius, which is suitable for allowing EDA nitrogen lone pair electrons to be used as soft Lewis bases. According to the findings, ZIF-8-EDA successfully removed Cd ^2+^ ions in an aqueous solution with high and selective selectivity compared to other metal ions. Evidence of this fact is its strong interactions with amino groups in the modified ZIF-8 accompanied by the formation of highly stable complexes.

### Adsorption isotherms, kinetics, and thermodynamics

Adsorption isotherms are important for describing how the molecules or ions of adsorbate interact with adsorbent surface sites; also, they are critical in optimizing the use of the adsorbent. The Langmuir and Freundlich adsorption isotherm models were used to evaluate the equilibrium data in the present study. The Langmuir isotherm suggests monolayer adsorption on a homogeneous surface without interaction between the adsorbed molecules and is given as follows^56^:

2 $${q}_{e}=\frac{{q}_{m}{K}_{L}{C}_{e}}{1+{K}_{L}{C}_{e}}$$

where q_e_ (mg/g) is the equilibrium Cd^2+^ ion concentration on the adsorbent, C_e_ (mg/L) is the equilibrium Cd^2+^ ion concentration in the solution, and q_m_ (mg/g) is the maximum adsorption capacity. K_L_ is the Langmuir adsorption constant (l/mg), relating the free energy of adsorption, and its high value indicates the high affinity of Cd^2+^ ions to the adsorption sites.

The Freundlich isotherm model proposes monolayer adsorption with a heterogeneous energetic distribution of active sites, accompanied by interactions between the adsorbed molecules, and is expressed as^57^:

3 $$q_{e} = K_{F} C_{e}^{{1/n}}$$

where K_F_ is a constant relating to the adsorption capacity and 1/n is an empirical parameter relating to the adsorption intensity, which varies with the heterogeneity of the material.

**Figure 12:**
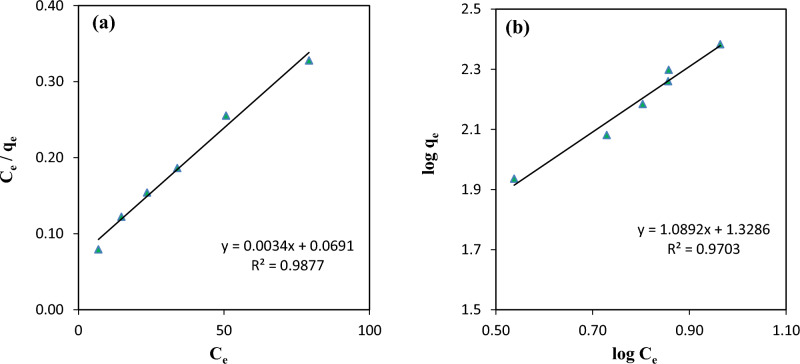
Langmuir (**a**) and Freundlich (**b**) isotherm plots for the adsorption of cadmium ions using ZIF-8-EDA.

The Langmuir and Freundlich model constants, along with the correlation coefficient (R^2^) values, are presented in Table 2. A comparison of the Langmuir and Freundlich isotherm models (Fig. 12) showed that the adsorption characteristics of Cd^2+^ ions onto ZIF-8-EDA followed more closely to the Langmuir isotherm equation. Therefore, it is noteworthy that ZIF-8-EDA has remarkable potential for removing Cd^2+^ ions from aqueous solution.

**Table 2:** Langmuir and Freundlich constants for the adsorption of cadmium ion using ZIF-8-EDA.

Langmuir model			Freundlich model		
q_m_ (mg/g)	K_L_ (L/mg)	R^2^	1/n	K_F_	R^2^
294.11	0.049	0.987	1.08	20.3107	0.970

In order to examine the controlling mechanism of the adsorption process, kinetic models are used to test the experimental data. The pseudo-first-order equation is one the most widely used rate equations to describe the adsorption of an adsorbate from the liquid phase. The pseudo-first order equation is generally expressed as follows^58,59^:

4 $${q}_{t}={K}_{e}(1-{e}^{-{k}_{1}t})$$

where q_e_ and q_t_ (mg/g) are the amount of adsorbed Cd^2+^ ions at equilibrium and at time t (min), respectively, and k_1_ is the pseudo-first-order rate constant (1/min).

The pseudo-second-order model is more suitable for describing the kinetic behavior of adsorption in which chemical adsorption is the rate-controlling step, and the equation is given as follows^60^:

5 $$\frac{t}{{q}_{t}}=\frac{1}{{k}_{2}{q}_{e}^{2}}+(\frac{1}{{q}_{t}})t$$

where k_2_ (g/mg.min) is the rate constant of the pseudo-second-order equation.

**Figure 13:**
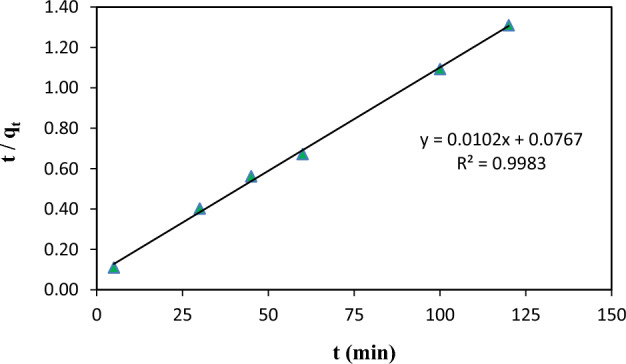
Plot of pseudo-second order kinetic models for the cadmium ion adsorption onto ZIF-8-EDA.

The rate constants for the pseudo-first-order and pseudo-second-order models and the correlation coefficient values are presented in Table 3. As can be seen, calculated correlations were closer to unity for the pseudo-second-order kinetic model; therefore, the adsorption kinetics could be estimated more favorably by the pseudo-second-order kinetics model rather than the pseudo-first-order kinetics for Cd^2+^ (Fig. 13). Thus, the chemical process controlled the overall adsorption rate of the Cd^2+^ ions through the sharing of electrons or by covalent forces via the exchange of electrons between the adsorbent and adsorbate.

**Table 3:** Kinetic parameters of pseudo-first order and pseudo-second order models for the sorption of cadmium using ZIF-8-EDA.

Pseudo-first-order kinetic model			Pseudo-second-order kinetic model		
k_1_(min^-1^)	q_e_ (mg/g)	R^2^	k_2_ (g/mg.min)	q_e_ (mg/g)	R^2^
0.0219	49.15	0.951	0.0021	98.03	0.998

The thermodynamic behavior of the Cd^2+^ ion adsorption by ZIF-8-EDA was investigated using thermodynamic parameters that included the change in free energy (∆G°), enthalpy (∆H°), and entropy (∆S°). These parameters are calculated from the following equations:

6 $${\text{ln}}{K}_{D}=\frac{{\Delta S}^{^\circ }}{R}-\frac{{\Delta H}^{^\circ }}{RT}$$

where K_D_ is the distribution coefficient (ml/g), ΔH° is the enthalpy change (kJ/mol), ΔS° is the entropy change (kJ/mol.K), T is the temperature (K), and R is the universal gas constant (8.314 J/mol.K). The Gibbs free energy change (ΔG°) is calculated from the following equation:

7 $${\Delta G}^{^\circ }={\Delta H}^{^\circ }-T{\Delta S}^{^\circ }$$

**Figure 14:**
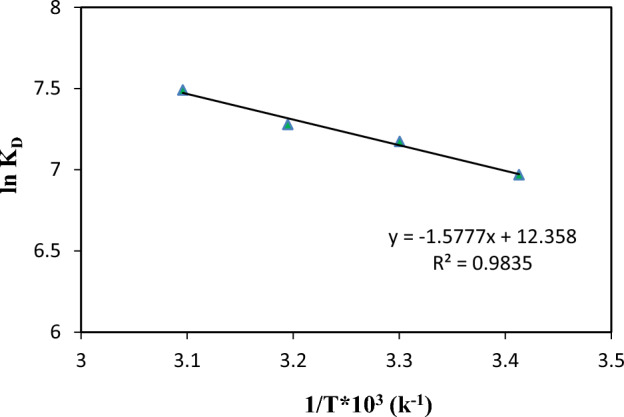
Plots of ln K_D_ vs. 1/T for the cadmium ion adsorption onto ZIF-8-EDA.

Based on Eq. 7, the ∆H° and ∆S° thermodynamic parameters could be calculated from the slope and intercept of the plot of ln K_D_ vs. 1/T, respectively (Fig. 14). These thermodynamic parameters are given in Table 4. It was found that free energy changes for physisorptions were generally between − 20 and 0 kJ/mol, the physisorption together with chemisorption were within − 20 to − 80 kJ/mol, and pure chemisorptions were in the range of − 80 to − 400 kJ/mol^61^. The calculated ΔG° values suggested that the adsorption processes of Cd^2+^ ions on the ZIF-8-EDA could be considered as physisorption together with the chemisorption process. Also, it was found that adsorption was the dominating system. The positive values of ∆H° confirmed the endothermic nature of the process. The positive ∆S° values indicated the affinity of the amino functionalized ZIF-8 for Cd^2+^ ions.

**Table 4:** Thermodynamic parameters for cadmium ion adsorption onto ZIF-8-EDA.

ΔG°(kJ/mol)				ΔH° (kJ/mol)	ΔS° (kJ/mol K)
20 °C	30 °C	40 °C	50 °C		
− 16.99	− 18.02	− 19.04	− 20.07	13.11	0.102

For the comparative objectives, the adsorption capacities of Cd^2+^ ion adsorption onto various adsorbents are listed in Table 5. As can be seen, ZIF-8-EDA had a good value of adsorption capacity in comparison with the other adsorbents. This indicated the strong adsorption characteristic of the synthesized adsorbent for removing Cd^2+^ ions as heavy metal cations.

**Table 5:** Comparison of maximum adsorption capacity (q_m_) for adsorption of Cd (II) using different adsorbents.

Adsorbent	q_m_ (mg/g)	References
Chitosan templated mesoporous silica	1.662	^62^
Polyamidoamine dendrimer grafted magnetic graphene oxide nanosheets	435.85	^63^
Thiol-functionalized metal organic framework material (HS-mSi@MOF-5)	98.0	^64^
Polyacrylic acid modified magnetic mesoporous carbon	406.6	^65^
Mungbean husk	34.85	^66^
Nano-hydroxyapatite	64.1	^67^
Sulfonic acid functionalized nonporous silica microspheres	178.8	^68^
MOF-DHz	188	^69^
(Fe_3_O_4_-ED)/MIL-101(Fe)	155	^70^
TMU-4	48	^71^
TMU-5	43	^71^
NH_2_-Zr-MOFs	177.35	^72^
Chitosan	150	^73^
TMU-16-NH_2_	126.6	^27^
Fe_3_O_4_@TAR	210	^74^
ZIF-8-EDA	294.11	This study

### Regeneration and reusability experiment

**Figure 15:**
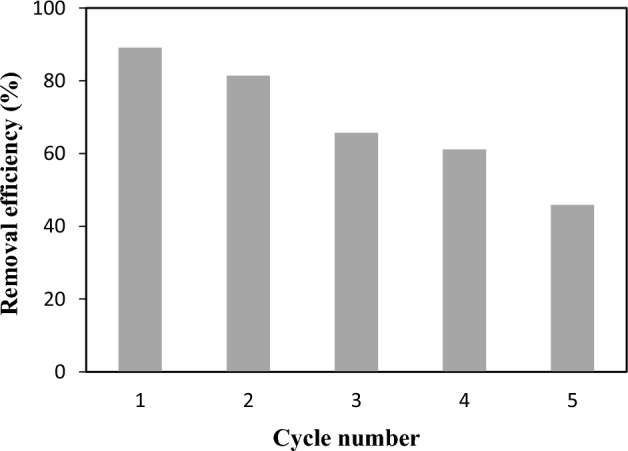
Reusability of ZIF-8-EDA for the cadmium ion removal.

The regeneration of the adsorbent is one of the factors used in assessing its potential for commercial applications. In order to improve the usability of ZIF-8-EDA, 0.1 M HCl was used as the desorbing agent in regeneration experiments to remove Cd^2+^ from a loaded adsorbent. In this condition, the H^+^ ions in the acid solution replaced the Cd^2+^ ions on the surface of the loaded ZIF-8-EDA adsorbent, making them into their chloride form. As shown in Fig. 15, after five regeneration cycles, the cadmium ion removal efficiency of the adsorbent decreased from 89.04 to 45.9% due to the irreversible partial occupation of the adsorbent active sites by H^+^ ions; however, even after five cycles, the adsorption of cadmium ions was still stable at around 45.9%, which indicated that the ZIF-8-EDA would be an effective adsorbent with low energy costs and without structural damage. Moreover, it had a high ability to be regenerated for removing Cd(II) from aqueous solutions.

## Conclusions

This study examined the synthesis and functionalization of ZIF-8 with ethylenediamine (EDA) and its subsequent use for the adsorption of ${Cd}_{\left(aq\right)}^{2+}$ ions. Post-synthetic modification is a useful technique that can be used to prepare new MOFs; it can also serve as an effective method for fine-tuning synthesized MOFs for fast and selective adsorption. ZIF-8-EDA was successfully applied to achieve the highly selective adsorption of Cd^2+^. It was found that ZIF-8-EDA's amino groups had enhanced coordination with Cd^2+^ ions. Furthermore, The HSAB theory states that Cd^2+^ ions have superior adsorption properties compared to Ni^2+^ and Pb^2+^ ions. The adsorption mechanism of the mentioned metal ions by the amino functional ZIF included electrostatic adsorption, chemical coordination due to their interaction with the non-bonding pair of electrons on the N atoms in the amine groups of the EDA, and ion exchange. More than 90% of cadmium ions were removed from the aqueous solution in the batch adsorption experiments at a [metal ion]_i_ of 50 mg/L, an adsorbent dosage of 0.05 g/L, and a pH of 6 after 60 min when using the amino-functionalization of zeolite imidazolate framework (ZIF-8-EDA). The findings demonstrated that ZIF-8-EDA's selective capture ability could be described by the order of Cd^2+^ > Pb^2+^ > Ni^2+^‏. It should be noted that this functionalization did not improve Ni^2+^ adsorption, and ZIF-8 had a higher performance for Ni^2+^ adsorption compared to ZIF-8-EDA. The experimental data fit the Langmuir equation well, with good correlation coefficients. The negative values of ΔG° indicated the feasibility of the spontaneous nature of the adsorption process. The positive values of ΔH° indicated that the adsorption process was endothermic. ZIF-8-EDA showed remarkable selectivity and regeneration efficiency for the adsorption of Cd(II). Furthermore, NH_2_-functionalization-ZIF-8 had an excellent adsorption capacity of 294.1 mg/g. Based on the findings, ZIF-8-EDA, with a high specific surface area and abundant active surface sites, could potentially serve as an efficient adsorbent of ${Cd}_{\left(aq\right)}^{2+}$ ions from wastewater.

## Data Availability

All data generated or analyzed during this study are included in this published article.
